# Enhancement of iodinin solubility by encapsulation into cyclodextrin nanoparticles

**DOI:** 10.1080/14756366.2017.1421638

**Published:** 2018-01-16

**Authors:** Anthony Prandina, Lars Herfindal, Sylvie Radix, Pål Rongved, Stein O. Døskeland, Marc Le Borgne, Florent Perret

**Affiliations:** a Université de Lyon, Université Claude Bernard Lyon 1, Faculté de Pharmacie – ISPB, EA 4446 Bioactive Molecules and Medicinal Chemistry, SFR Santé Lyon-Est CNRS UMS3453 – INSERM US7, Lyon Cedex, France;; b Department of Pharmaceutical Chemistry, School of Pharmacy, University of Oslo, Oslo, Norway;; c Centre for Pharmacy, Department of Clinical Science, University of Bergen, Bergen, Norway;; d Department of Biomedicine, University of Bergen, Bergen, Norway;; e Université de Lyon, Université Claude Bernard Lyon 1, Institut de Chimie et Biochimie Moléculaires et Supramoléculaires, UMR 5246 CNRS – CPE Lyon – INSA, Villeurbanne Cedex, France

**Keywords:** Iodinin, solubility, amphiphilic α-cyclodextrin, nanoparticles, encapsulation

## Abstract

Phenazine is known to regroup planar nitrogen-containing heterocyclic compounds. It was used here to enhance the bioavailability of the biologically important compound iodinin, which is near insoluble in aqueous solutions. Its water solubility has led to the development of new formulations using diverse amphiphilic α-cyclodextrins (CDs). With the per-[6-desoxy-6-(3-perfluorohexylpropanethio)-2,3-di-*O*-methyl]-α-CD, we succeeded to get iodinin-loaded nanoformulations with good parameters such as a size of 97.9 nm, 62% encapsulation efficiency and efficient control release. The study presents an interesting alternative to optimizing the water solubility of iodinin by chemical modifications of iodinin.

## Introduction

Phenazine is a dibenzo annulated pyrazine present in many natural products^1–3^ and has become the parent substance of many synthetic bioactive molecules^4–6^. The broad spectrum of biological activities of phenazine explains the success of research programs exploiting this scaffold. The most striking examples are the targeting of antibiotic-tolerant bacterial biofilms and *Mycobacterium tuberculosis* by halogenated phenazines^7^. Other derivatives such as endophenazine G showed activity against community-associated methicillin-resistant *Staphylococcus aureus*^8^. Phenazine-1-carboxylic acid derivatives exhibit fungicidal activities^9^ and finally numerous phenazines were developed as anti-cancer agents^10^, for example, the novel pyrano[3,2-*a*]phenazine derivatives demonstrated antiproliferative activity against the HepG2 cancer cell line^11^.

**Figure 1.**
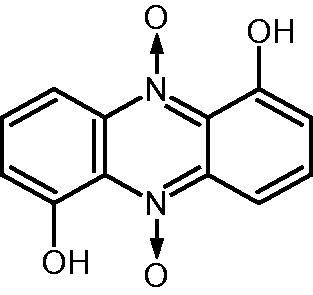
Structure of iodinin (1,6-dihydroxyphenazine 5,10-dioxide).

Iodinin (Figure 1) was first discovered in 1939^12^ within *Chromobacterium iodinum* bacterial cultures. In 1943, McIlwain demonstrated its anti-streptococcal action^13^. For the last 75 years, iodinin has been isolated from diverse soil bacteria (e.g. *Brevibacterium iodinum*^14^, *Pseudomonas phenazinium*^15^, *Nocardiopsis dassonvillei*^16^, and *Acidithiobacillus ferrooxidans*^17^), or marine bacteria (e.g. *Actinomadura* sp.^18^, *Streptosporangium* sp.^19^). Recently, recombinant *Pseudomonas* strains were used successfully to propose an alternative for the biosynthesis of natural phenazines^20^. Iodinin displays other biological activities, including anti-microbial and cytotoxic properties^21^^,^^22^. Actually, it is worth noting that iodinin showed remarkable selective toxicity to acute myeloid leukaemia (AML) and acute promyelocytic leukaemia (APL) cells, with various proposed mechanisms of action suggested such as DNA intercalation and activation of apoptotic signalling proteins (e.g. caspase-3)^19^.

The first total synthesis of iodinin was recently described by Viktorsson et al.^21^. The physical chemical properties of iodinin can be summarised as follows: it is a dark red solid, stable in acidic solution, unstable in alkali. Iodinin’s solubility in different solvent can be summarised as follows: it is soluble in benzene, toluene, xylene, carbon disulphide, chloroform, ethyl acetate, THF, concentrated sulfuric acid, glacial acetic acid and sodium hydroxide. It is also slightly soluble in hot alcohol. In parallel, iodinin is practically insoluble in cold alcohol, ether, acetic acid, petroleum ether, or amyl alcohol^21^. Finally, iodinin is absolutely insoluble in water. In addition, various assays^21^ showed that iodinin solutions turned (i) pink when it was solubilised in most solvents; (ii) purple in chloroform with formation of crystals with a coppery sheen; (iii) red in glacial acetic acid and (iv) brilliant blue in sodium hydroxide with the deposition of green crystals from unstable sodium derivatives. It thus appears that iodinin is a bioactive molecule, which is difficult to manage in most biological investigations. To overcome this issue, we envisaged to complex iodinin with cyclodextrins (CDs) to increase aqueous solubility and bioavailablility.

Amphiphilic CD derivatives have been available for decades^23^^,^^24^ mainly to overcome problems of native CDs that limit their applications in pharmaceutical fields. Indeed, since dissociation takes place too readily upon dilution, untimely release may take place during administration to the patient, so that inclusion complexes inside simple water-soluble CD appear ineffective for drug delivery applications. In fact, the use of amphiphilic CDs (i) enhances the interaction with biological membranes, (ii) modifies or enhances interaction of CDs with hydrophobic drugs, and (iii) allows self-assembly of CDs, forming nanosized carriers and encapsulating drugs^25^^,^^26^. Polycationic CD nanoparticles containing siRNA have been recently used for the delivery of siRNA to the glomerular mesangium^27^.

Our group has published several studies demonstrating synthesis of amphiphilic CDs which were able self-assemble to form stable nanoparticles. Most of our amphiphilic derivatives have been prepared by modifying their primary face with hydrocarbon or perfluorocarbon lipophilic chains^28–31^. As demonstrated previously for a hydrophobic indeno[1,2-*b*]indole analog^32^, not only could these nanoparticles encapsulate this CK2 inhibitor but also released it in a controlled manner.

This study deals with the formation and anti-leukemic activity of iodinin-loaded nanoparticles made from amphiphilic α-CDs. Encapsulation efficiency and release profiles are reported and show the beneficial effect of the fluorinated amphiphilic α-CD derivatives. The non-toxicity of these derivatives on red blood cells confirmed their potential use for *in vivo* assays.

## Experimental

### General

**Figure 2.**
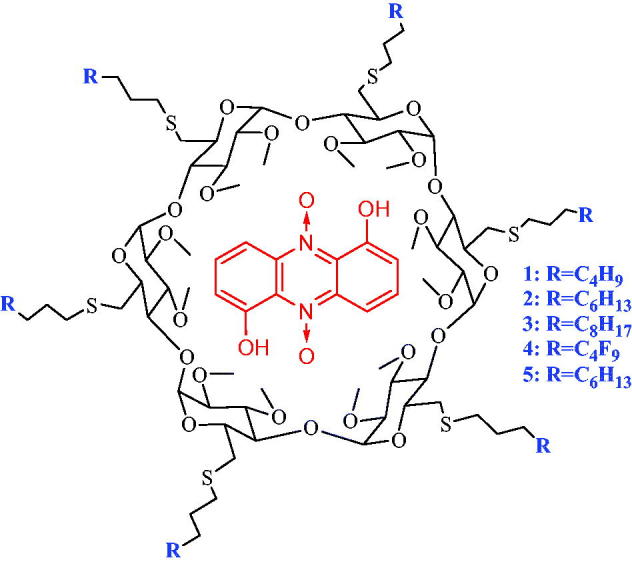
Structure of inclusion complex of iodinin (red) in amphiphilic alkyl (**1**–**3**) or perfluoroalkyl (**4**,**5**) α-cyclodextrins.

All chemical were purchased from Sigma-Aldrich, La Jolla, CA, USA and were used without further purification. Native α-cyclodextrin was generously provided by Roquette Frères (Lestrem, France). Amphiphilic fluorinated α-CDs and their hydrocarbon analogues (Figure 2) were synthesised as previously described^28^^,^^31^. Briefly, after the selective protection of the primary hydroxyl groups with tertbutyldimethylsilyl groups, all the secondary hydroxyl groups were methylated using sodium hydride and methyl iodide. Removal of the tertbutyldimethylsilyl groups was performed with tetrabutylammonium fluoride in THF and introduction of the methanesulfonyl groups with methanesulfonyl chloride. Finally, the hydrophobic chains (fluorinated or hydrocarbonated) were introduced by nucleophilic substitution of the leaving groups by the thiolate derivate, generated *in situ* by the basic hydrolysis of the 3-perfluoroalkylpropane (or alkyl) isothiouronium salts using cesium carbonate. The structures and purities were confirmed using ^1^H and ^13^C NMR and mass spectroscopy analysis.

Iodinin was isolated from batch cultures of the bacterium *Streptosporangium* sp. The bacterial mass culturing conditions, as well as the protocol for DMSO-extraction, subsequent HPLC-purification and identification of iodinin by MS and NMR were carried out as previously described^19^^,^^22^.

Dynamic light scattering measures were performed using a Zetasizer Nano ZSP instrument from Malvern Instruments, Malvern, UK.

### Preparation of nanoparticles by the highly loaded method

The iodinin loaded nanoparticles based α-CD were prepared by the nanoprecipitation technique, using a 0.8 × 10^−4 ^M solution of preformed (1:1) iodinin:α-CD complexes overloaded with an additional amount of iodinin in the THF phase. The total concentration of iodinin was 1.6 × 10^−4 ^M (iodinin/CD ratio = 2). The relevant solution of the preformed complex in THF (25 ml, 1 day stirring) was poured drop-wise into deionised water (50 ml) while stirring. A slightly turbid emulsion of nanospheres spontaneously formed. Solvent and a part of water were evaporated under reduced pressure and the total volume adjusted to 50 ml with water.

### Particle size measurements

The mean particle size (diameter, nm) and the polydispersity index (PDI) of nanospheres were measured by dynamic light scattering using a NanoZS instrument, which analyses the fluctuations of scattered light intensity generated by diffusion of the particles in a diluted suspension (dynamic light scattering data are shown in Figures S1–S5 and Zeta potentials of empty and loaded nanoparticle dispersions are presented in Figures S6?S10). The measurements were carried out at 25 °C. Experiments were performed in triplicate.

### Determination of the encapsulation efficiency

For measuring the loading efficiency, after the formation of nanoparticle suspensions by the highly loaded method, non-encapsulated iodinin in the nanoparticle dispersions was separated by centrifugation at 50,000 rpm for 1 h in order to settle down the loaded nanoparticles. The supernatant was removed. The precipitate was then lyophilised overnight, and the resulting powder containing the loaded nanoparticles was dissolved in a known amount of THF in order to obtain a clear solution. The absorbance of supernatant and THF solutions was analysed using an UV spectrophotometer at 289 nm to calculate the encapsulated drug quantity. Loading capacity was expressed in terms of associated drug percentage:

$$Associated\;drug\;\left(\%\right)=\frac{\left[determined\;iodinin\;quantity\;(mol)\right]}{\left[initial\;iodinin\;quantity\;(mol)\right]}\times 100$$

### *In vitro* release studies

The suspensions of nanoparticles made from C_6_H_13_, C_8_H_17_ and C_6_F_13_ derivatives loaded with iodinin (1 ml of a 0.8 × 10^−4 ^M solution) were introduced into a dialysis tube (cutoff 5000 Da) at 25 °C. This tube was then placed in a higher volume (20 ml) of phosphate buffered solution (pH 7.4) for a period of time. Same experiments have been done with non-encapsulated iodinin by using 1 ml of a 0.8 × 10^−4 ^M iodinin THF/water solution. Aliquots of 1 ml of the buffered solution were removed at different time intervals to calculate the proportion of released and encapsulated molecules by UV spectrometry at 289 nm.

### Cytotoxicity studies

The formulations were tested on the Brown Norwegian myeloid leukaemia (BNML) rat-derived AML cell line IPC-81^33^. The cells were cultured in Dulbecco’s Modified Eagles Medium (DMEM; Sigma, La Jolla, CA, USA) enriched with 10% horse serum (Invitrogen, Carlsbad, CA, USA), and added 100 IU/L penicillin and 100 mg/L streptomycin (both from Cambrex, Verviers, Belgium), and cultured in a humidified atmosphere (37 °C, 5% CO_2_). For cytotoxicity testing, the cells were seeded in 96 well tissue culture plates at 150,000 cells/mL. The cells were exposed to various concentrations of empty or iodinin-loaded nanoparticles for 24 h and then fixed in 2% buffered formaldehyde (pH 7.4) with the DNA-specific dye Hoechst 33342 (Polysciences Inc., Eppelheim, Germany) and scored for apoptosis as previously described^34^^,^^35^.

## Results and discussion

### *α-*CD *nanoparticles*

It has been reported that the highly loaded method was the most efficient for encapsulating hydrophobic compounds inside amphiphilic CD-based nanoparticles^30^. Since iodinin is hydrophobic, this method was chosen for its encapsulation, using THF as co-solvent which allowed the solubilisation of both iodinin and amphiphilic CD derivatives.

**Figure 3.**
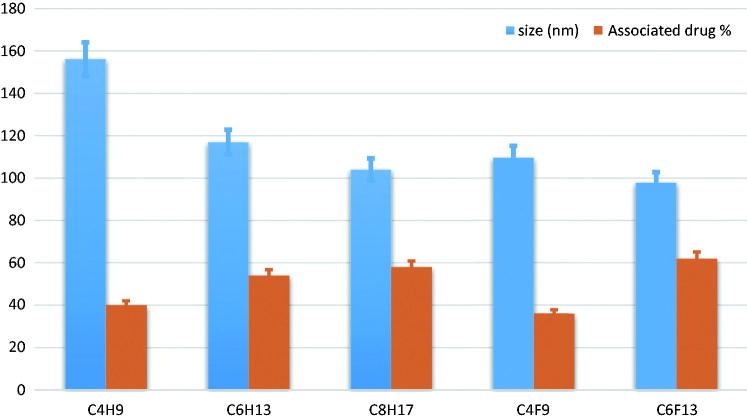
Sizes (in nm) of loaded nanoparticles and percentages of encapsulated iodinin for each derivative.

As shown in Table 1 and Figure 3, the different nanoparticles had similar sizes, ranging from 97.9 nm to 156.2 nm. Comparing **1/4** and **2**/**5**, it was noticed that, for the same hydrophobic chain length, perfluorinated nanoparticles gave lower diameters than the hydrogenated ones. In fact, the specific properties of fluorous chains allowed for a more compact organisation of the hydrophobic chains inside the nanoparticles. Furthermore, for the same series (hydrogenated or fluorinated), it was an inverse relationship between the chain length and the size of the nanoparticle. It is also worth noting that empty nanoparticles had similar sizes as the loaded nanoparticles. All these data were found to match findings previously described in literature^31^^,^^32^.

**Table 1.** Characteristics of loaded nanoparticles made from amphiphilic α-cyclodextrins.

Derivative	Side chain	Nanoparticle size (nm) loaded/*empty*	Polydispersity index (PDI) loaded/*empty*	Associated drug (%)
**1**	C_4_H_9_	159.3*/162.3*	0.07*/0.04*	40
**2**	C_6_H_13_	117.0*/126.3*	0.10*/0.13*	54
**3**	C_8_H_17_	104.1*/105.8*	0.11*/0.05*	58
**4**	C_4_F_9_	109.7*/120.7*	0.02*/0.25*	36
**5**	C_6_F_13_	097.9*/90.6*	0.08*/0.10*	62

The experiments, run in triplicate, yielded particles with narrow size distribution (PDI <0.2) demonstrating high homogeneity of the nanoparticle suspensions.

The loading efficiency of iodinin in these various nanoparticles ranged from 36% to 62% for C_4_H_9_ and C_6_F_13_, respectively. Nevertheless, unlike what has been observed previously for acyclovir, nanospheres made from fluorinated α-CDs did not have significant impact on the encapsulation rate. The main differences were observed by varying the chain length (40%, 54% and 58% for C_4_H_9_, C_6_H_13_ and C_8_H_17_, respectively), suggesting that C_8_F_17_ would be slightly more efficient for encapsulation of iodinin.

**Figure 4.**
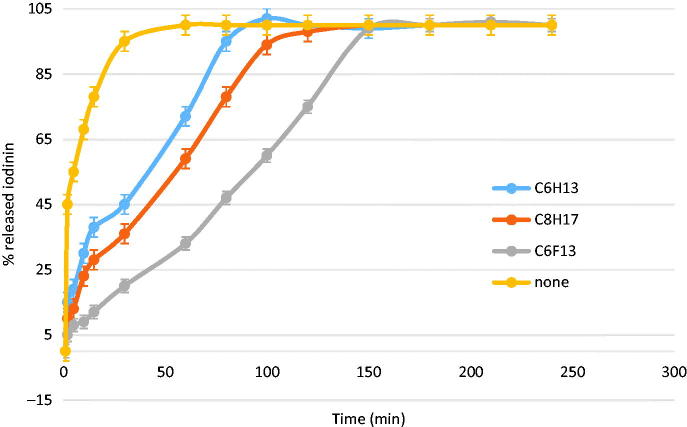
Release profiles of iodinin in phosphate buffered aqueous solution (pH 7.4).

The controlled release studies was performed on suspensions having at least 50% encapsulated iodinin (i.e. C_6_H_13_, C_8_H_17_ and C_6_F_13_) in comparison with the profile obtained without any nanoparticles (a 0.8 × 10^−4 ^M iodinin solution alone in THF/water solution). As shown in Figure 4, in the absence of nanospheres, the concentration equilibrium between the outside and inside compartments of the dialysis tube was obtained in less than 40 min.

The release profiles showed the positive effect of the nanoparticles on the controlled release (Figure 4). Iodinin release from highly loaded nanospheres reached completion within more than one hour for hydrocarbon amphiphilic α-CDs and between 2.5 and 3 h for the fluorinated nanospheres. After 1 h, 72% of the encapsulated iodinin were released from the C_6_H_13_ nanospheres *versus* only 30% from the fluorous analogue. It can be explained by the fact that fluorinated chains enhance intermolecular interactions inside the supramolecular assemblies compared to hydrogenated analogues, leading to more stable nanoparticles. These observations confirm previous studies which have showed that nanoparticles based on fluorinated compounds delayed acyclovir release, showing their potential for applications to drug delivery^30^.

**Figure 5.**
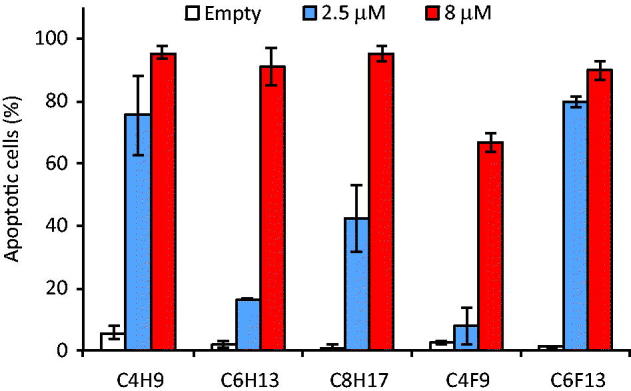
Cytotoxicity of iodinin-loaded amphiphilic CD-nanospheres towards the AML cell line IPC-81. The cells were incubated with the different formulations for 24 h before assessment of cell death. The data are average of two separate experiments. The error bars indicate the two measurements.

A particular point needs to be added about the toxicity of amphiphilic α-CDs. A recent study^36^ related a study of cytotoxicity on red blood cells. The results confirmed the potential of amphiphilic α-CDs to formulate bioactive molecules and then to be used for in vivo assays. We tested empty or iodinin-loaded fluorinated amphiphilic CD nanospheres for ability to induce cell death in the BNML-derived rat AML cell line IPC-81. This cell line produces AML with typical signs of the disease in xenograft mouse models, and responds to the benchmark AML drug daunorubicin *in vitro* and *in vivo*^37^. We found no toxicity towards the IPC-81 cells with the any of the empty nanoparticles (Figure 5). Iodinin-loaded nanoparticles, however, efficiently induced IPC-81 AML cell death within 24 h (Figure 5). From the different CD-compositions tested, we found that the C_4_H_9_ and C_6_F_13_ were the most potent formulation, whereas C_6_H_13_ and C_4_F_9_ were the least potent formulations. This is opposite to what was seen in the release studies, which showed that C_6_H_13_ released their cargo at a faster rate than C_6_F_13_ (Figure 4). This suggests that internalisation of the nanoparticles indeed play a role in the cytotoxic effect of the amphiphilic α-CD nanospheres. Although the efficacy of the nanospheres appeared lower than the original compound^19^^,^^21^, the encapsulation of iodinin is expected to lower toxic effects on non-target cells, thus increasing the therapeutic index for this potent AML-selective compound.

## Conclusions

This study describes the successful preparation of iodinin-loaded nanoparticles. The results indicate that nanoencapsulation of iodinin in α-CDs by the highly loaded method is possible, without any additional surface-active agent. With per-[6-desoxy-6–(3-perfluorohexylpropanethio)-2,3-di-*O*-methyl]-α-CD we were able to perform the most loaded nanoparticles (% of associated drug = 62) with a size of 97.9 nm. Tests of these nanoparticles on AML cells showed that they were efficient inducers of cell death, due to the encapsulated iodinin, since empty nanoparticles showed no adverse effects on the cells. Furthermore, amphiphilic α-CD derivatives could be functionalised on the secondary hydroxyl groups by targeting moieties such as folate^38^ or by incorporating the fragment antigen-binding (Fab) of a monoclonal antibody onto CDs to target IL-3 receptor α-chain (IL-3Rα, highly expressed on AML LSCs)^24^.

## Supplementary Material

IENZ_1421638_Supplementary_Material.pdf
