# Supplementary material for: Enhancement of iodinin solubility by encapsulation into cyclodextrin nanoparticles
Source: J Enzyme Inhib Med Chem. 2018 Jan 16;33(1):370–5. doi: 10.1080/14756366.2017.1421638 (PMC6009883; doi:10.1080/14756366.2017.1421638)
Supplement: IENZ_1421638_Supplementary_Material.pdf [file IENZ_A_1421638_SM3803.pdf]

Supplementary materials

Enhancement of iodinin solubility by encapsulation into cyclodextrin nanoparticles

Anthony Prandina[a,b], Lars Herfindal[c], Sylvie Radix[a], Pål Rongved[b], Stein O. Doskeland[d], Marc Le Borgne[a], and Florent Perret[e]

[a]Université de Lyon, Université Claude Bernard Lyon 1, Faculté de Pharmacie - ISPB, EA 4446 Bioactive Molecules and Medicinal Chemistry, SFR Santé Lyon-Est CNRS UMS3453 -INSERM US7, 8 avenue Rockefeller, F-69373, Lyon cedex 8, France; [b]Department of Pharmaceutical Chemistry, School of Pharmacy, University of Oslo, PO Box 1068 Blindern, N0316 Oslo, Norway; [c]Centre for Pharmacy, Department of Clinical Science, University of Bergen, Jonas Lies vei 87, N-5009 Bergen, Norway; [d]Department of Biomedicine, University of Bergen, Jonas Lies vei 91, N-5009 Bergen, Norway; [e]Université de Lyon, Université Claude Bernard Lyon 1, Institut de Chimie et Biochimie Moléculaires et Supramoléculaires, UMR 5246 CNRS - CPE Lyon - INSA, 43 Boulevard du 11 Novembre 1918, F-69622 Villeurbanne cedex, France.

Dynamic light scattering measures were performed using a Zetasizer Nano ZSP instrument from Malvern Instruments, UK.

			Size (d.nm):	% Intensity:	St Dev (d.nm):
Z-Average (d.nm):	159,3	Peak 1:	171,2	100,0	46,43
PdI:	0,069	Peak 2:	0,000	0,0	0,000
Intercept:	0,932	Peak 3:	0,000	0,0	0,000
Result quality :	Good				

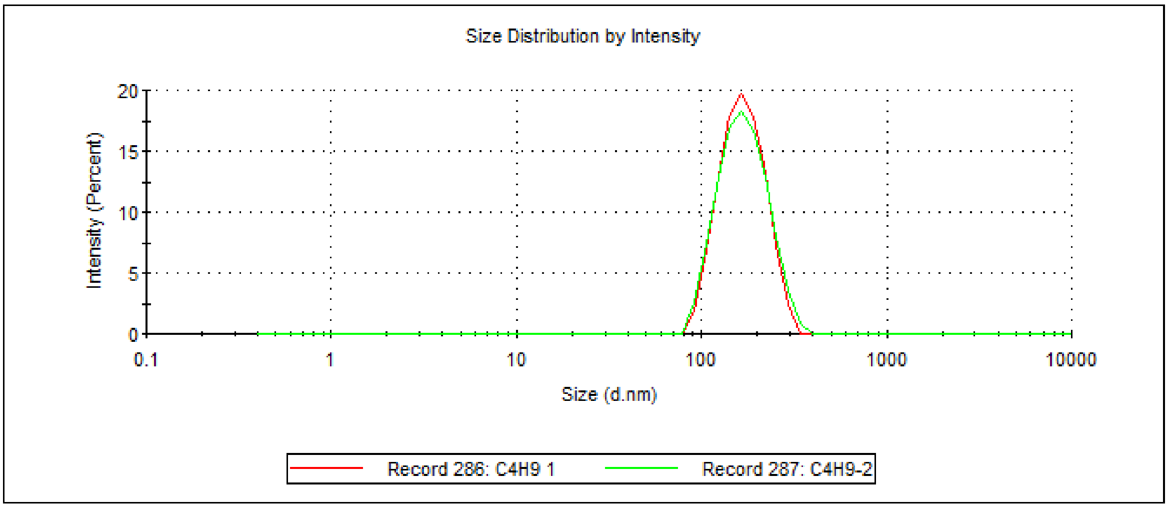

Figure S1. Dynamic light scattering experiments spectra and mean diameter of iodinin loaded C_4H_9 amphiphilic CDs.

			Size (d.nm):	% Intensity:	St Dev (d.nm):
Z-Average (d.nm):	117,0	Peak 1:	130,6	100,0	45,04
PdI:	0,096	Peak 2:	0,000	0,0	0,000
Intercept:	0,963	Peak 3:	0,000	0,0	0,000
Result quality :	Good				

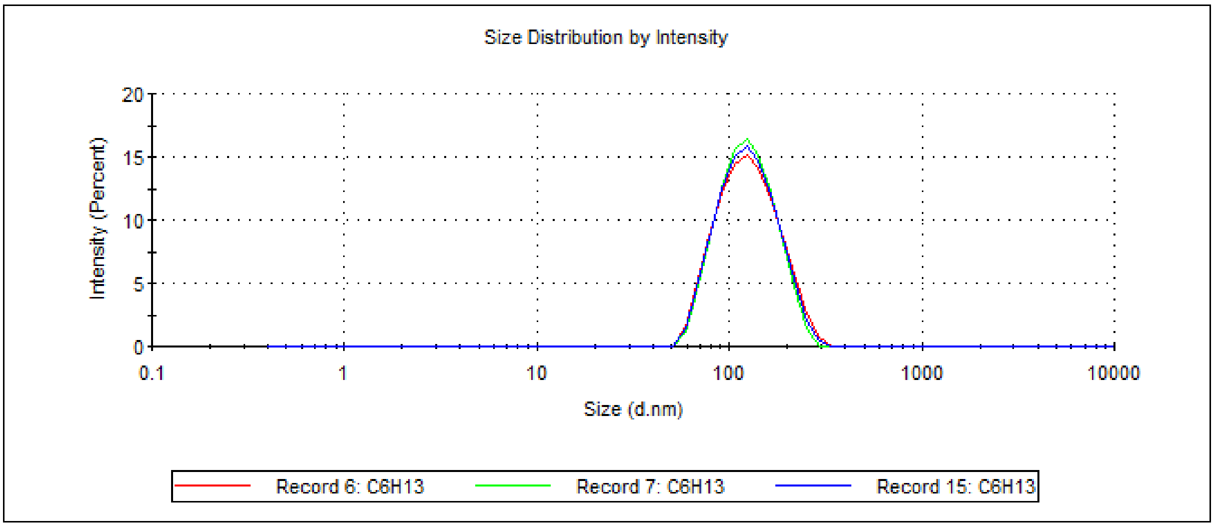

Figure S2. Dynamic light scattering experiments spectra and mean diameter of iodinin loaded C_6H_{13} amphiphilic CDs.

			Size (d.nm):	% Intensity:	St Dev (d.nm):
Z-Average (d.nm):	104,1	Peak 1:	116,1	99,5	44,22
PdI:	0,111	Peak 2:	4824	0,5	713,1
Intercept:	0,955	Peak 3:	0,000	0,0	0,000
Result quality :	Good				

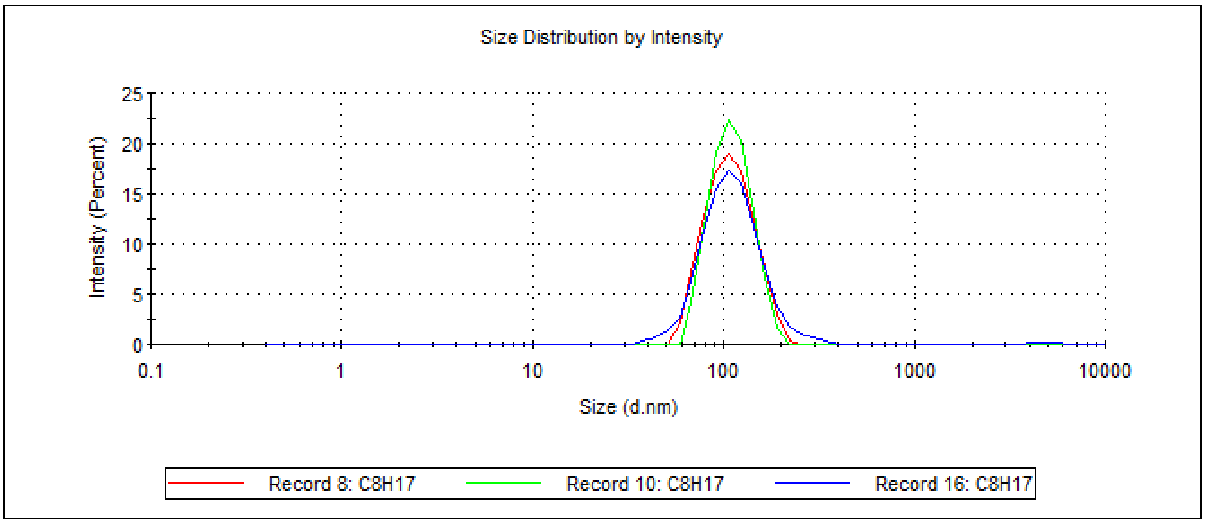

Figure S3. Dynamic light scattering experiments spectra and mean diameter of iodinin loaded C_8H_{17} amphiphilic CDs.

			Size (d.nm):	% Intensity:	St Dev (d.nm):
Z-Average (d.nm):	109,7	Peak 1:	114,6	100,0	26,27
PdI:	0,022	Peak 2:	0,000	0,0	0,000
Intercept:	0,952	Peak 3:	0,000	0,0	0,000
Result quality :	Good				

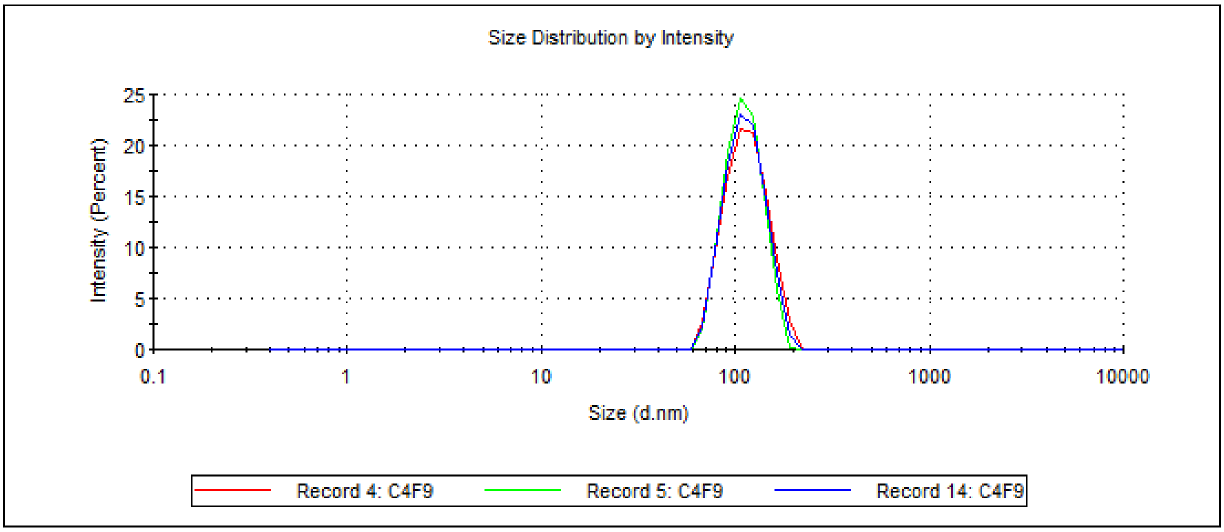

Figure S4. Dynamic light scattering experiments spectra and mean diameter of iodinin loaded C_4F_9 amphiphilic CDs.

			Size (d.nm):	% Intensity:	St Dev (d.nm):
Z-Average (d.nm):	97,89	Peak 1:	105,9	100,0	30,47
PdI:	0,075	Peak 2:	0,000	0,0	0,000
Intercept:	0,957	Peak 3:	0,000	0,0	0,000
Result quality :	Good				

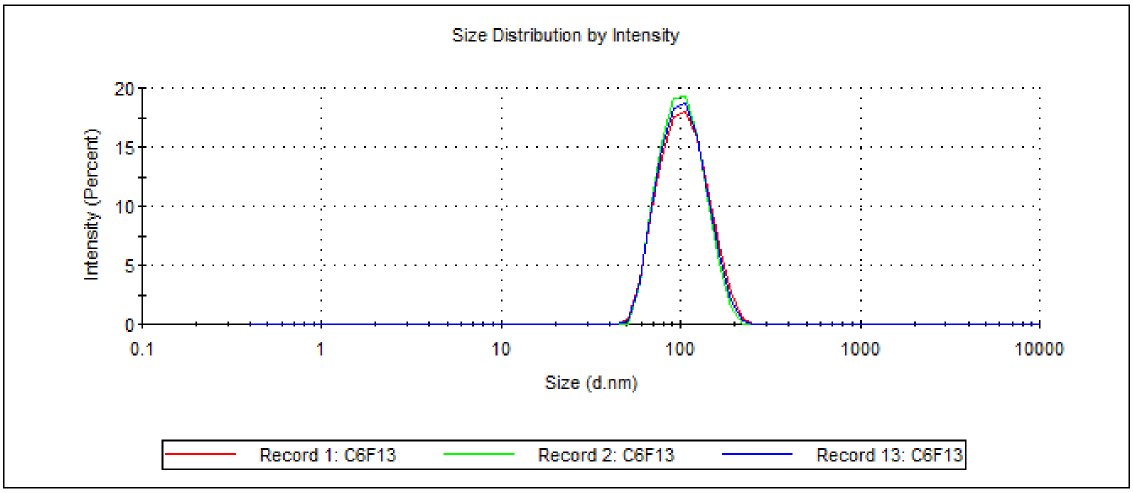

Figure S5. Dynamic light scattering experiments spectra and mean diameter of iodinin loaded C_6F_{13} amphiphilic CDs.

Zeta Potential Analysis: Zeta potentials of empty and loaded nanoparticle dispersions were measured by Malvern Zetasizer Nano-ZS (Malvern Instruments, UK) at 25°C, in triplicate to assess the surface charge. Zeta potential of nanoparticles were directly measured on suspension of nanoparticles in water pH 7.4.

	**Zeta potential (mV)**<br>Loaded / *Empty*
**C_4H_9**	-20.2 mV ± 3.5 / *-16.4 mV ± 4.0*
**C_6H_{13}**	-29.6mV ± 5.1 / *-25.1 mV ± 5.5*
**C_8H_{17}**	-43.3 mV ± 6.2 / *-38.0 mV ± 5.2*
**C_4F_9**	-26.6 mV ± 4.2 / *-21.9 mV ± 5.6*
**C_6F_{13}**	-32.5 mV ± 5.0 / *-28.0 mV ± 6.8*

No significant changes were observed for zeta potential values. For both empty and iodinin-loaded nanoparticles, they were in the same range with a little increase with the chain length. No differences were observed between loaded and empty ones. It is worth noticing that even if the amphiphilic cyclodextrins are not charged, the zeta potentials are negative at this pH, meaning that these suspensions are stable, avoiding aggregations. Recent results in DLS showed indeed that these suspensions were stable after 6 months (same sizes…). We found that the iodinin-loaded nanospheres using C_4H_9 and C_6F_{13} were the most potent formulations (highest cell death on AML cell line IPC-81), with zeta potentials of -20.2 mV and -32.5 mV, respectively. With the formulations using C_6H_{13} and C_4F_9 (with similar zeta potentials), cytotoxicity results were lower (Figure 5). Cytotoxicity and zeta potential were not correlated in this study.

			Mean (mV)	Area (%)	St Dev (mV)
Zeta Potential (mV):	-20,2	Peak 1:	-20,2	100,0	3,47
Zeta Deviation (mV):	3,47	Peak 2:	0,00	0,0	0,00
Conductivity (mS/cm):	0,0287	Peak 3:	0,00	0,0	0,00
Result quality :	Good				

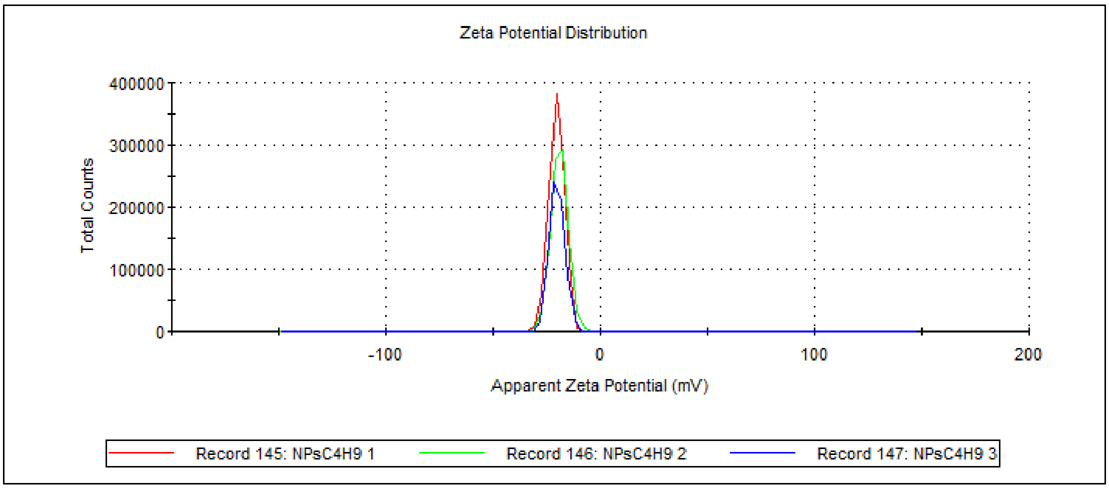

Figure S6. Zeta potential distribution of iodinin-loaded C_4H_9 amphiphilic CDs.

			Mean (mV)	Area (%)	St Dev (mV)
Zeta Potential (mV):	-29,6	Peak 1:	-29,6	100,0	5,09
Zeta Deviation (mV):	5,09	Peak 2:	0,00	0,0	0,00
Conductivity (mS/cm):	0,0346	Peak 3:	0,00	0,0	0,00

Result quality : Good

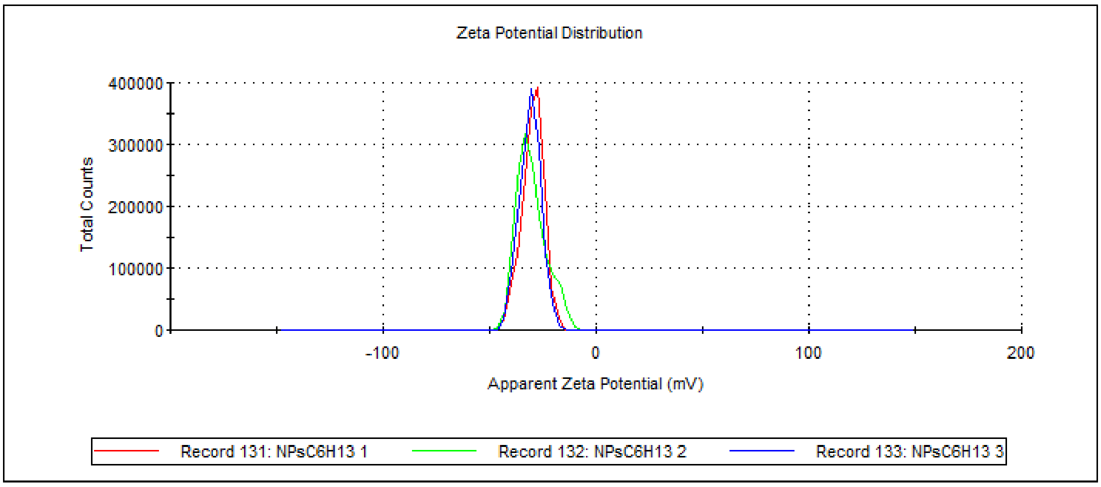

Figure S7. Zeta potential distribution of iodinin-loaded C_6H_{13} amphiphilic CDs.

			Mean (mV)	Area (%)	St Dev (mV)
Zeta Potential (mV):	-43,3	Peak 1:	-43,3	100,0	6,17
Zeta Deviation (mV):	6,17	Peak 2:	0,00	0,0	0,00
Conductivity (mS/cm):	0,0862	Peak 3:	0,00	0,0	0,00

Result quality : Good

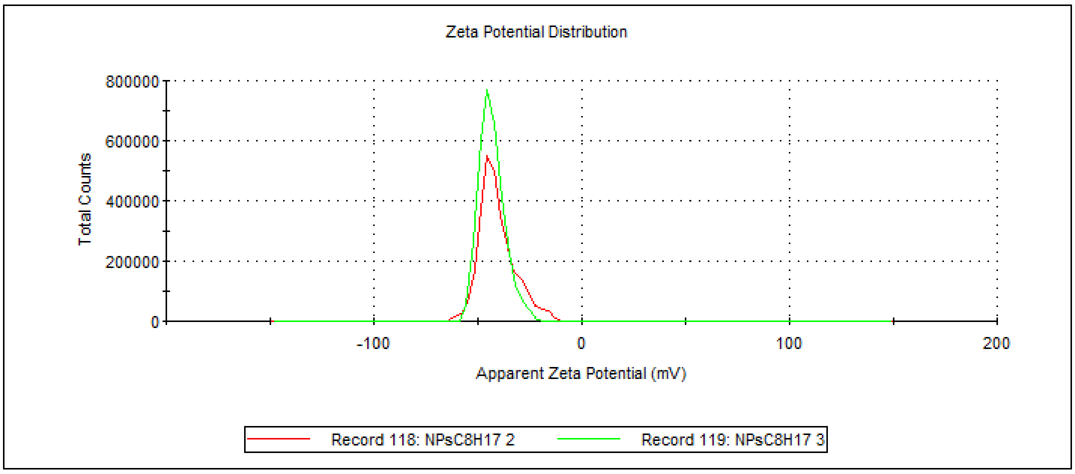

Figure S8. Zeta potential distribution of iodinin-loaded C_8H_{17} amphiphilic CDs.

			Mean (mV)	Area (%)	St Dev (mV)
Zeta Potential (mV):	-26,6	Peak 1:	-26,6	100,0	4,18
Zeta Deviation (mV):	4,18	Peak 2:	0,00	0,0	0,00
Conductivity (mS/cm):	0,0370	Peak 3:	0,00	0,0	0,00

Result quality : Good

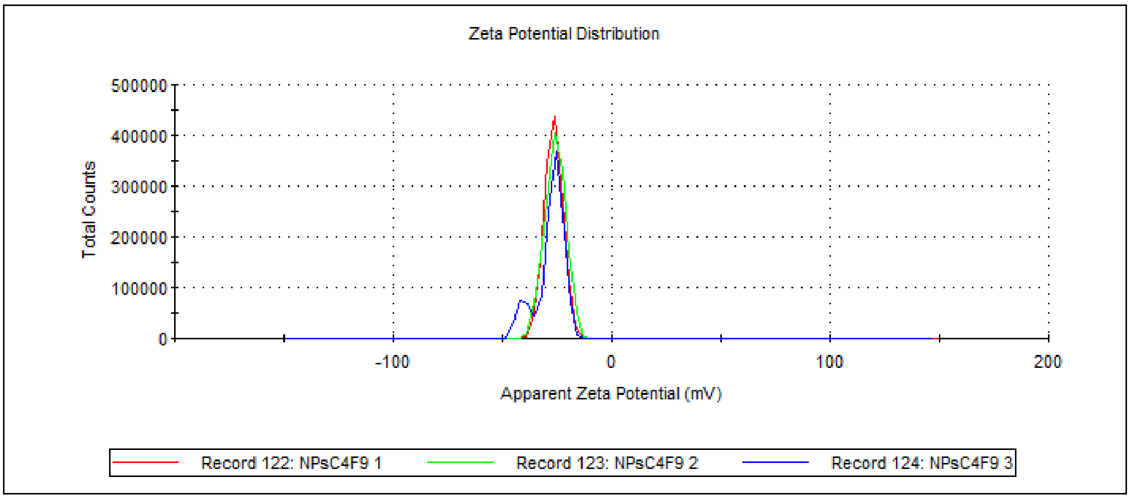

Figure S9. Zeta potential distribution of iodinin-loaded C_4F_9 amphiphilic CDs.

			Mean (mV)	Area (%)	St Dev (mV)
Zeta Potential (mV):	-32,5	Peak 1:	-32,5	100,0	5,00
Zeta Deviation (mV):	5,00	Peak 2:	0,00	0,0	0,00
Conductivity (mS/cm):	0,0506	Peak 3:	0,00	0,0	0,00

Result quality : Good

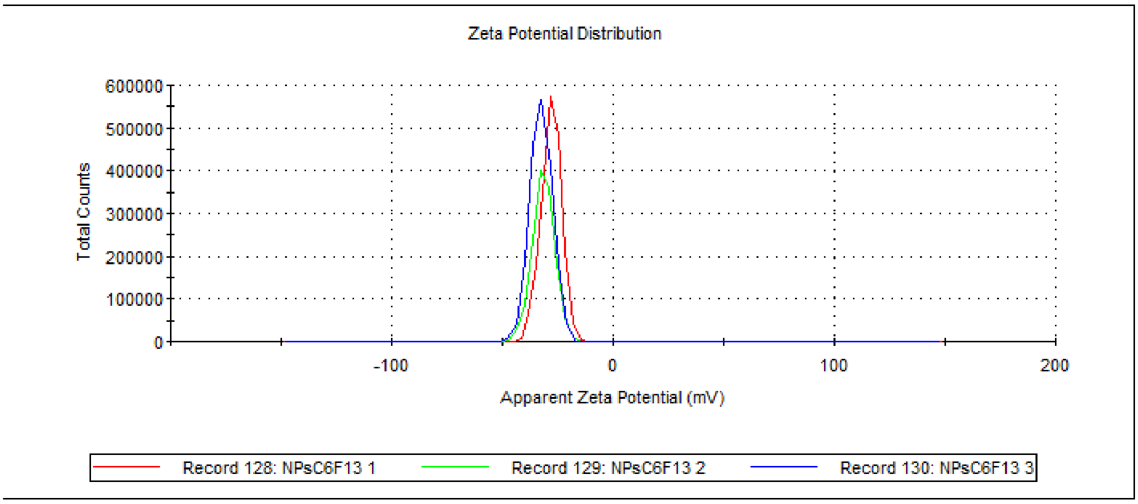

Figure S10. Zeta potential distribution of iodinin-loaded C_6F_{13} amphiphilic CDs.
